# Struggling with Discrimination: An Intersectional Exploration of Why Unequal Social Relations Persist in Residency Training Programs

**DOI:** 10.5334/pme.1961

**Published:** 2026-02-10

**Authors:** Justin T. H. Lam, Ryan J. Giroux, Han Yan, Adelle R. Atkinson, Abhaya V. Kulkarni, Christopher R. Forrest, Maria Athina (Tina) Martimianakis

**Affiliations:** 1 Department of Paediatrics, University of Western Ontario, Toronto, ON, CA; 2 Children’s Hospital, London Health Sciences Centre, Toronto, ON, CA; 3 Centre for Education Research and Innovation, Toronto, ON, CA; 4 Wilson Centre for Research in Education, University of Toronto, Toronto, ON, CA; 5 Department of Paediatrics, University of Toronto, Toronto, ON, CA; 6 Women & Children’s Health Program, St. Michael’s Hospital, Unity Health Toronto, Toronto, ON, CA; 7 Department of Family and Community Medicine, University of Toronto, Toronto, ON, CA; 8 Department of Paediatrics, University of Toronto, Hospital for Sick Children, Toronto, ON, CA; 9 Clinician Educator with the Royal College of Physicians and Surgeons of Canada, CA; 10 Division of Neurosurgery, Department of Surgery, University of Toronto, Hospital for Sick Children, Toronto, ON, CA; 11 Division of Plastic, Reconstructive and Aesthetic Surgery, Department of Surgery, University of Toronto, Toronto, ON, CA; 12 Department of Paediatrics, University of Toronto, Hospital for Sick Children, Toronto, ON, CA; 13 Wilson Centre for Research in Education, University of Toronto, Toronto, ON, CA

## Abstract

**Introduction:**

Although postgraduate medical education programs in North America have committed to addressing equity, diversity, and inclusion (EDI) issues, learning environment inequities persist and negatively impact learning outcomes. We conducted an exploratory qualitative study to identify mechanisms contributing to ongoing discrimination experiences.

**Methods:**

We took an intercategorical intersectional approach to conduct a qualitative thematic analysis that focused on unequal social relations as the unit of analysis. Participants were recruited from paediatrics, neurosurgery, and plastic surgery residency training programs. We performed multi-level inductive and deductive coding of semi-structured interviews informed by intersectionality and systemic and aversive racism.

**Results:**

13 participants were interviewed. Participants identified their social identities as marginalized or dominant, and reported intersectional exclusion experiences related to the former that disadvantaged them compared to their peers. Persistent unequal social relations could be mechanistically accounted for by aversive or systemic racism regardless of intersectional identities. Aversive discrimination appeared through performative EDI commitments, microaggressions, and social exclusion. Systemic discrimination contributed to exclusion stemming from the operationalization of cultural norms such as scheduling, social events, and censorship of taboo topics. Participants described variably successful compensatory strategies to overcome their marginalization.

**Discussion:**

Systemic and aversive discrimination contribute to reproducing unequal social relations. Evidence-based approaches exist to address these mechanisms and make learning environments fairer.

Despite advances in education research and teaching to make learning environments fairer, some postgraduate medical learners (residents) continue to disproportionately struggle with discrimination and harassment [1,2,3,4]. The ongoing discrimination faced by residents with intersectionally marginalized social identities in North America [5,6,7,8,9,10,11,12] suggests that there are underlying mechanisms of exclusion that remain under-addressed. In the Canadian context, specific accreditation standards require that clinical learning environments are supportive of all learners [13]. Failing to understand and address why these experiential inequities persist threatens resident wellbeing and belonging, increases burnout and attrition, and erodes trust in educational institutions.

Intersectional studies in residency education to date have taken an *intra-*categorical or “multiple intersections” approach to intersectionality to show that trainees with two or more marginalized identities experience double or triple jeopardy [14]. These discrimination experiences based on intersectionally marginalized identities undermine learner experiences of belonging, hamper professional identity formation, and deter them from certain specialities [15,16,17]. For example, studies that apply this theoretical approach highlight how trainees who are female and Black [18,19], racialized and LGBTQ [20,21], female and under-represented based on other identities [22], or female, Arab, and internationally trained [23,24] all struggle with different training challenges compared to non-marginalized peers.

Intra-categorical intersectional studies add to the literature by fostering a more nuanced understanding of under-representation within the medical profession [25,26,27,28] compared to studies using standpoint approaches that attribute individual experiences to specific single identities [29,30,31]. Specifically, intra-categorical studies highlight how a learner’s experience does not hinge on a single identity, and that sharing a social identity does not imply shared experiences [32]. An intra-categorical intersectional lens appreciates how a Black female learner can experience both racism and sexism, or how an internationally-trained male learner from a low socioeconomic status (SES) background can experience discrimination based on xenophobia and classism. Intra-categorical intersectional studies generate targeted evidence to inform educational interventions that provide equitable opportunities and advance fairness in learning environments, emphasizing that programs should know their learners’ intersecting identities.

Institutions have responded to uncontested evidence that racism, sexism, and classism impact learner experiences for the better part of the last decade. However, as intra-categorical intersectional studies have shown, students continue to experience exclusion and discrimination despite said efforts, and learners with two or more marginalized identities continue to experience multiple jeopardy [33]. There is a clear need to understand why such discrimination persists. One area for further attention is to understand how systems might contribute to ongoing experiences of discrimination. For example, the Foucauldian concept of normalization highlights how dominant notions of professional identity are rooted in the experiences of white men. Constructed this way, professional identity entrenches a racial and gendered hierarchy within the medical profession that disadvantages women even in female-dominated fields, as well as others with marginalized backgrounds.

Therefore, in contrast to the typical *intracategorical* approaches described above, we chose an *intercategorical* approach, which shifts the focal point of the intersectional analysis from learner experiences to the very mechanisms for how unequal social relations are systemically reproduced. In this way, we can add to the literature by clarifying how systemic phenomena, stemming from ideologies such as racism, sexism, or classism, continue to manifest at the individual level [28]. This intercategorical intersectional approach could identify organizational interventions that would potentially increase a sense of belonging for more students than current standardized approaches. This kind of intersectional analysis has been theorized within medical education [27,28,29,34,35], but empirically, primarily explored outside of medical education literature [31,36,37,38,39,40], and is a useful analytic frame for understanding how unequal social relations are “produced, experienced, reproduced, and resisted in everyday life [28].”

We applied an intercategorical intersectional approach to explore the specific socio-cultural mechanisms by which unequal social relations in residency programs are reproduced at an individual level. To keep the study contextually focused, we interviewed residents from the same medical school at one teaching hospital across three training programs to understand what mechanisms of discrimination impacted their lived experiences. Identifying these mechanisms for both inclusion and exclusion experiences are critical if training programs are to meaningfully address learning environment EDI issues and ensure alignment between organizational priorities, learner experiences, and patient care.

## Methods

### Theoretical framing

Our study was conducted within a critical constructivist paradigm, meaning that we employed theoretical concepts that attuned us to power and its effects, including that knowledge and truth are socially constructed. We operationalized an *intercategorical* approach focused on relationships of inequality as the unit of analysis [31]. In the sociological intersectionality literature, this approach has also been described as a process centred model of intersectionality that “places primary attention on context and comparison at the intersections as revealing structural processes organizing power” [38]. For example, an intercategorical approach to intersectionality revealed how the learning of 7^th^ grade boys in a Danish classroom was variably impacted by complex social relations of exclusion and inclusion that manifested in gender and ethnic identity negotiations [37]. This intercategorical study highlights how a boy becomes ethnicized as deviant compared to a boy with a “normal” European ethnic identity, and how the former’s attempts to regain status by performing a hypermasculine gender identity is also eventually labelled as deviant, ultimately reinforcing his subordinate status. The study is intercategorically intersectional in that it shows how the boys negotiate becoming a member of either a majority or minority ethnic or gender grouping, and how these processes compensate or overshadow one another in perpetuating unequal classroom social relations [37].

In our study, we similarly stayed attuned to the intercategorical negotiations as well as to the structural and systemic mechanisms that contributed to instances of inclusion and exclusion in our study context. This application of the intercategorical approach allowed us to focus on how the process of being racialized, gendered, or otherwise discriminated against based on identity categories is dynamic, negotiated, and impacts resident training experiences at different times for different individuals, regardless of their intersecting identities [38]. Exploring experiences at one teaching hospital allowed us to distill how each participant’s interconnected identities mediate their individual experience in a way that reflects macro level systems of privilege and oppression that find expression in routine organizational practices [28,29]. Finally, consistent with an intercategorical approach, we also describe the experiences that participants linked to what they perceived to be dominant social identities in their work contexts [36,39,41].

#### Sampling

In keeping with a critical constructivist, intercategorical approach, we sampled broadly for participants with both marginalized and dominant identities instead of only recruiting participants with social identities at specific neglected intersections [36,39,41]. An open call went to all residents in paediatric, neurosurgery, and plastic surgery residency training programs at a large urban centre medical school. This selection of training programs was both theoretical and convenient: theoretical in that sampling both medical and surgical training programs allows for analysis of differences that either cut across or distinguished different programs and/or professional cultures; convenient in that these programs had expressed a shared interest in studying this phenomenon. Participants self-selected to participate. We also took a snowball approach and asked participants to recommend colleagues they thought would bring relevant perspectives. Recruitment occurred from April 2021 to October 2022. This study was approved by the University of Toronto and Hospital for Sick Children’s Research Ethics Boards.

#### Data Collection

Interviews were conducted with 13 participants. We used an identity exercise as a reflective primer for the interviews, which broadly explored participant identities, their perceived relevance to work and training, how participants’ attributed disadvantage or advantage to their different social identities, how they experienced those identities as mediating their training experiences compared to their peers, and potential avenues for reform. General observations from each interview were shared with the research team and informed iterative refinement of the interview guide. Importantly, interviews were conducted by research team members without conflicts of interest, pre-existing supervisory relationships with participants, or other roles that could foreseeably affect participant comfort and safety.

To ensure adequate sample size and rigour for our study, we used the concept of information power [42]. A number of research practices amplified the richness of our data and our capacity to identify patterns of exclusion and inclusion, thereby ensuring we had sufficient information to answer our research question with a smaller sample of participants. First, we used semi-structured interviews together with an elicitation device. We maintained a tight study focus, interviewing participants from one teaching hospital at a single medical school. We also used the reflection tool to focus our dialogue around participant identities, probed in interviews to elicit rich descriptions of participant experiences, and conducted longer interviews to increase dialogue quality. For example, interviews lasted between 60 to 90 minutes. Second, we relied on a methodological approach that combined deductive and inductive analysis, thus ensuring that we did not miss important insights derived from the uniqueness of the life stories of participants. Third, we used a recognized theoretical approach and the concept of intersectionality throughout the design and analysis, integrating additional theoretical concepts in the analysis as needed to make sense of participant experiences [42].

#### Positionality and Reflexivity

The research team was made up of individuals with different intersectional backgrounds from different training programs and levels of training which included: being racialized, white, or white-passing, model minority status, cisgender and/or a member of LGBTQ2S community, low socioeconomic status background, religious and ethnic minority, and Métis ancestry and/or immigrant/settlers on Canadian territories. This allowed the research team to conduct research with, for, and alongside learners from marginalized backgrounds, consistent with an intersectional approach [29]. The research team took a reflexive approach to this research project by discussing their own positionality using the same identity reflection exercise that was used for participants during the study’s design, data collection, and analysis phases. The group considered how their own social identities related to experiences of inclusion and exclusion in their training, clinical practice, and teaching experiences to appreciate and mitigate potential blind spots in their analysis of participant experiences. For example, we always considered an experience of inclusion or exclusion from multiple situated identities to see if the experience was unique to a participant because of their intersecting identities or could also apply to a single identity category.

#### Data Analysis

We used intersectionality as an analytic lens to conduct a qualitative thematic analysis [43]. Our goal was to explore how different social and professional identities related to the awareness, experiences, and mechanisms that mediated participant perceptions of social relations within training. Procedurally, initial coding of interview transcripts was completed by two research team members who did not have clinical supervisory roles and could access all transcripts (O.F., M.A.M.). Initial codes were discussed with the entire research team and refined. An anonymized coding summary was then shared with the two lead clinical members of the research team for a second and third layer of inductive then deductive analysis (J.L., R.G.). All layers of analysis were then shared with the entire team for review, discussion, and finalization. Differences in analysis were discussed and were considered opportunities to enrich meaning making from the situated perspective of each research member. While we also paid attention to participant experiences that were unique to their training program, our tightly focused sampling approach (one medical school, same teaching hospital) led to participant accounts of practices that were more similar than different and thus our dataset was not conducive to a comparison of structural and socio-cultural differences between the different training programs. We thus focused the intersectional analysis on the reproduction of systemic inequities through experiences of inclusion and exclusion that cut across training program experiences.

To perform an intersectional analysis, each participant was assigned a code. We then coded the social identities that each participant reported and coded for how participants attributed advantage or disadvantage to each of their identities as well as the training experiences that they associated with each identity. We also coded for how participants attributed advantage or disadvantage to their peers’ perceived dominant or marginalized social identities to track unequal social relations. We began with an inductive layer of coding to identify common themes regarding exclusion or inclusion experiences, and the ways that those experiences were unique or not to particular intersectional identities. A second layer of inductive analysis, sensitized by an intercategorical approach to intersectionality, coded for mechanisms that maintained the unequal social relations noted by participants using their reports of differential treatment compared to their peers as a starting point. Through this second layer of analysis, we identified systemic and aversive racism as mechanisms for participant experiences. A third layer of deductive analysis using the concepts of systemic and aversive racism was conducted to identify further examples and identify nuanced examples within the data while accounting for participants’ intersectional identities.

### Conceptual Framing for Analysis

Systemic or institutional racism describes forms of racism that are embedded within standard procedures, written or unwritten policies, and habitual everyday practices. As a result, these policies and procedures may not appear racist on the surface but ultimately have a racist effect and directly or indirectly result in experiences of exclusion or discrimination by racialized people. Systemic racism is found in the existing culture and structure of an organization and produces and reproduces a system of unequal relations that causes differential harm for racialized trainees [44,45]. For example, systemic racism within the medical profession, as a reflection of systemic racism in Canadian society, has contributed to the historic under-representation of Indigenous and Black physicians in the medical profession [46,47,48].

Aversive racism occurs when people who usually endorse and act in concordance with egalitarian values discriminate (often unintentionally) against people from historically marginalized groups when faced with ambiguous situations or unclear guidelines [49,50]. These actions are rationalized or justified based on factors other than race in ways that reflect interlocking systems of privilege and oppression. Moreover, these actions are often the result of normal cognitive processes such as in-group favoritism, social dominance, and implicit bias [49,50]. Social dominance theory states that society and social systems have at least two groups – a dominant one that have attributes and resources deemed valuable by society, and one or more less-dominant groups. Implicit bias is the unconscious, automatic association of negative stereotypes or attitudes with a particular group. In group favoritism is when individuals act in ways that favor members of one’s own group over outsiders [49,50]. For example, microaggressions can be a downstream effect of aversive racism because they result from implicit biases and because their perpetrators are often unaware of their discriminatory impact.

Aversive racism and systemic racism are also conceptually linked in that aversive racism is one way of understanding an interpersonal manifestation of systemic racism stemming from the normative culture of a particular context. Systemic racism is embedded within the sociocultural fabric of an organization and shows up as routine habits, practices, and policies that influence how individuals evaluate excellence and competence, distribute promotions and awards, or select leaders. In short, systemic racism socializes individuals to certain implicit biases that downstream have an aversive racist effect on racialized learners.

## Results

Our intercategorical intersectional analysis revealed two key findings. First, participants related their exclusion experiences to one or more of their intersectionally marginalized social identities and reported how these experiences contributed to unequal social relations compared to the perceived educational experiences of their more conventional peers. Second, the maintenance of these unequal social relations could be accounted for mechanistically as instances of systemic or aversive discrimination. Through our analytical approach, we show how the sociomaterial effects of ideologies such as racism, sexism, and classism contribute to the intercategorical intersectional experiences of exclusion reported by participants. We conclude with a description of how participants experienced unequal social relations in an intersectional way, with specific examples of how they were racialized, gendered, and marginalized, and the compensatory strategies involved in their struggle with discrimination.

Thirteen participants self-selected to be part of the study and were interviewed. Although we did not specifically recruit for students with intersectionally marginalized identities, all participants identified two or more social identities that they felt were non-dominant in their training environment. Identities that participants reported experiencing as marginalized included being Muslim or member of a religious minority, LGBTQ, racialized as non-White, international medical graduate, low socioeconomic status, and young age. Ten of thirteen participants were pediatric residents (Table 1). Notably, twelve of the thirteen participants identified as women.

**Table 1:** Participant Demographics.


**Residency Training Program**	Number (total n = 13)

Paediatric	10

Surgical	3

**Gender**	

Female	12

Male	1

**Other Non-Dominant Social Identities (n)** Muslim (5) LGBTQ (2) Black (2) East Asian (2) South and Southeast Asian (7) Middle Eastern (2) International medical graduate (3) Low socioeconomic status (3) Age (younger or older) (4) PGY1-5	

Reported Dominant Social Identities (identified by participants; * if also held by some participants) Christian (1) Heterosexual* (6) White* (1) Upper socioeconomic status* (1) Able-bodied* (2) Relatives in Medicine (1) The counts in this section only apply to those held by participants. The counts do not include dominant social identities that were attributed to non-participant peers in interviews.	


Even though we asked participants about both inclusion and exclusion experiences, participants predominantly focused on the latter during accounts of their training. Participants readily identified their own social identities that they felt were dominant or marginalized in their training environment. All participants reported struggling with marginalization based on their non-dominant social identities. All participants contrasted their marginalization with the perceived relative advantage of their peers who were identified as having more privileged, visible social identities, such as presenting as male, white, or Canadian trained (Table 1). These experiences of differential treatment led participants to feel excluded and frustrated, even though none of the participants shared experiences of overt discrimination.

### Aversive Discrimination as a Mechanism

Participants reported how the performativity of formal program EDI commitments, microaggressions related to their non-dominant identities, and social exclusion from dominant groups perpetuated unequal social relations. Through our analysis, these experiences could be mechanistically explained by aversive discrimination.

### Performative Program Commitments

Participants experienced a mismatch between performative program commitments to EDI and their own struggles with exclusion and discrimination. Our participants did not report perceived malicious intent but nevertheless reported how this misalignment made them feel lesser than their peers.

*“I find that in medicine, people are so educated that they feel that they’re immune to these kinds of things, or they would never do something [discriminatory]. So, [my supervisors and peers] never check themselves because they think that they’re above it…and no one ever says anything to them, so they continue going on perpetuating these microaggressions.”* – Participant 6

*“We can’t be in an ivory tower when it comes to EDI… there needs to be some explicit way of connecting what EDI is on the floor or in practice, and EDI research and theory.” –* Participant 4

*“You get a lot of emails, but you don’t see a lot of action… our program sends emails [asking] how we can support you. But it should be like, we want to make changes to our program that embraces the diversity of our resident group, please let us know which of the following could be helpful.” –* Participant 10

Aversive racism could be a contributing mechanism in these experiences of exclusion, as participants reported their supervisors and peers felt that they were egalitarian and bias-free and were unaware when they acted on their implicit biases, inflicted microaggressions, or demonstrated in-group favoritism.

### Microaggressions

Participants also reported struggling with feeling lesser than their peers with dominant identities due to microaggressions related to their marginalized identities. Our participants noted that these microaggressions from supervisors, colleagues, and patients stemmed from implicit biases which they attributed to align with normalized constructs of doctors as white and male.

*“I feel like I have to work harder to seem like a better doctor to my patients because I’m a woman, and because maybe I’m a woman who is not white”* – Participant 4, related to being a racialized woman

*“Definitely, 110%, people take my male colleagues more seriously than me, so that’s frustrating, of course…Sometimes I feel I have a less leeway to express frustration than my white colleagues because I always have this fear that I’m an angry black woman” –* Participant 6, related to being a racialized woman

Participants were keenly aware of being held to a different standard compared to their peers when it came to being perceived as experts. Some participants also reported experiencing stereotype threat, worrying that they might fulfill a negative stereotype based on their non-dominant identities such as the angry black woman like Participant 6 reported above, the quiet woman of colour, or the less knowledgeable internationally trained resident [51]:

*“I feel that people in medicine prioritize a Type A personality or a very aggressive or confident person… I have felt that I need to be more assertive, even though that’s not really my personality. Just because of the culture of medicine.” –* Participant 2, related to being a racialized woman

*“Like if I answer a question, I don’t have a Canadian accent, [I’m] non-white. Even if a white Canadian answers a question and its wrong it might still be acceptable as, ‘Okay, maybe just think of it this way instead’…But if someone like myself answers it wrong it may be more harped on, like, ‘That’s wrong, where did you learn that? It’s different here’” –* Participant 11, related to being a racialized, internationally trained man

*“I think when I came to Toronto as an international medical graduate, I certainly felt from my co-residents that, because I had trained internationally, I wasn’t as good. It was very palpable from the get-go that they didn’t respect international medical graduates” –* Participant 8, related to being a racialized, internationally trained woman

Participants felt lesser than their peers because they were being mistaken for lower status professions and clerical staff in ways that their peers were not. In the examples below, in addition to sometimes being mistaken for a member of the allied healthcare team, participants were also mistaken for a unit clerk or secretary, a role that is stereotypically held by racialized women in the study context.

“*Sometimes I reflect…did that interaction not go as well because of what I said or the way that I presented the material? …or did this interaction go that way because I’m a brown young female?…I don’t even get mistaken as a nurse…people think I’m a unit clerk or a secretary.” –* Participant 10, related to a racialized woman

*“I had worn my hair curly that day…And [my supervisor] was just like, ‘Every time I see you your hair is looking more and more wild. What, are you applying for an admin role?’…This bothered me because you’re now relating the appearance of my hair to an administrative role. It was quite offensive and unprofessional”* – Participant 12, related to being a racialized woman

Participants also reported struggling with being mistaken for a patient’s parent or family member or for other trainees because of a shared marginalized identity in ways that were not happening to their peers with more dominant identities.

*“The physician [mistook] me for one of his fellows…He kept being like, “Oh, do you remember when we saw that patient?”… No one said anything…it was so obvious what was happening, and I felt humiliated even though obviously it wasn’t my fault… I feel our professional relationship was ruined after that. I think if somebody had said something, I wouldn’t have had to bear that burden.” –* Participant 6, related to being a racialized woman

Aversive racism, an active mechanism in these examples, could account for how participants were made to feel lesser than their peers through the layering of implicit biases that resulted in professional and personal miscategorization based on their intersectionally marginalized identities. Aversive racism is a plausible mechanism given that participants related these microaggressions to implicit biases that their colleagues and supervisors seemed unaware of inflicting.

### Exclusion from Dominant Social Groups

Participants also experienced exclusion from social groups that privileged dominant attributes, such as being white, upper SES, Canadian trained, heterosexual, and able-bodied that made them feel excluded by their peers.

*“A lot of residents are in these long-term heterosexual relationships, and so, people just assume that I have a husband, or a boyfriend, or a fiancé…in those situations, I feel like a little bit of an outsider… I think sometimes people just make assumptions because that’s the most common situation.” –* Participant 1, based on sexual orientation

*“I feel the international residents maybe slightly get sort of left out, particularly if they are very few in number. I was lucky that in my program we had two or three other international residents.” –* Participant 3, based on racialized and Muslim identities

To our study participants, the role that their own non-dominant social identities played in this exclusion was clear, but as described in the aversive racism literature, it was never the stated reason for why this social exclusion from their residency year cohort occurred.

*“There was a picture from someone’s birthday and it was most of our residency cohort…except for the people who are ethnic minorities… And then more of these [social media] posts of them all posting the same video or pictures or wedding invites… It was just this tight-knit, White, rich group… there definitely is an element of choosing people who are alike in race and status that isn’t recognized by that group.”* – Participant 5, based on low SES, racialized identities

*“I basically formed all of my friendships outside of my residency program. It was kind of hostile…to a point where it’s not particularly pleasant…every time I thought that I was getting on well with someone, a third party would tell me that they had said something unpleasant about me.”* – Participant 8, based on international training background

These experiences could be accounted for mechanistically by the psychological processes that drive aversive discrimination such as social dominance and in group favoritism.

### Systemic Discrimination as a Mechanism

Participants reported exclusion experiences related to how cultural norms were integrated into program policies and practices by default. In particular, social events, scheduling, and the perceived censorship of certain topics in the learning environment were experienced by participants as exclusionary based on “the ways things were”. In other words, the operationalization of normative assumptions grounded in the local, default medical culture that participants reported as predominantly white, Eurocentric, Christian, and heteronormative, which can be mechanistically accounted for by systemic discrimination

Default social events that involved drinking alcohol were one example of systemic discrimination identified in this study. Multiple participants struggled with exclusion from these social events and related these experiences to their marginalized religious background. Participants reflected how other residents from other visible and non-visible marginalized backgrounds also shared similar experiences, and perceived this to have a discriminatory effect that perpetuated unequal social relations amongst residents.

Participants who identified as racialized Muslim men or women, for example, shared:

*“I’ve spoken to a couple people in my program who felt excluded [from social events]…because they didn’t fit the common culture or felt uncomfortable…[or] don’t drink for other reasons…[such as] a white person who was Catholic by background but did not drink… [When planning a social] someone jumped in to say that we should also send recipes out for mocktails….a couple people laughed it off, saying no, they can just take out the alcohol” –* Participant 7

*“I think as a Muslim person…I felt like a lot of [social] things had drinking … I didn’t always feel like it was inclusive.” –* Participant 9

*“Your culture, the way you socialize, is different and I felt isolated initially… like I don’t drink alcohol, so if those social events are at a bar I probably would not be too keen on attending.” –* Participant 11

Participants also related their marginalized social identities to having to expend extra effort compared to their peers to celebrate holidays or observe religious practices that were more meaningful to them, such as Eid, Friday prayers, or lunar new year.

*“I had reached out to my program, weeks in advance, to tell them that Eid is coming up, I don’t know which day it will be because it depends on moon sighting. I was very worried because it’s hard to get time off, but the program was very, very receptive.” –* Participant 7

*“…[we] don’t want to miss Eid, but living in the west we always miss it because it’s not a recognized holiday or you feel you can’t ask for the time off… we have Friday prayers which are usually our mandatory day for praying… I don’t know how many people know that that’s a requirement”* – Participant 11

*“You get an email that says Happy Chinese New Year or Happy Eid or Happy Ramadan, but they don’t think about how to actually include these residents or to make accommodations for them, which is annoying.”* – Participant 10

Participants were grateful for protected holiday time but struggled with the additional burden compared to their peers to celebrate holidays that were more meaningful given their intersectional social identities.

Participants also shared how they noticed educational practices that reinforced the idea of a neutral medical culture [52,53,54], which meant that certain topics, such as Islamophobia and anti-Muslim racism, Middle Eastern politics, and experiences related to being from a lower SES, were silenced or perceived as taboo conversations.

*“There’s always a power differential between a resident and a staff, so if a staff says their opinion about political systems in the Middle East, even if I don’t agree with the staff, I have to play it really careful not to offend that staff… a non-Canadian or minority resident might not be able to express their opinions.”* – Participant 3, related to having racialized, Muslim, and non-citizen identities

*“It’s so hard to condemn the killing of innocent lives and you can’t even say that is wrong. For me that was hypocrisy because you’re able to say that Black Lives Matter but you can’t even say the word Palestine.” –* Participant 11, related to having racialized Muslim identities

*“What I have found disappointing is that co-residents… will advocate and speak about issues that are more palatable…but not speak out about things that are a bit more risky…In medicine, I just didn’t belong overall…It’s really a cultural thing, thinking that being neutral is somehow a thing that is possible” –* Participant 9, related to having racialized Muslim identities

This perceived censorship that resulted from systemic racism and classism within medical culture was reported to contribute to feelings of exclusion and the preservation of unequal social relations in the learning environment relative to their peers, who did not have to face similar struggles.

### The intercategorical intersectional nature of discrimination

Overall, our participants reported feeling disadvantaged compared to their peers due to their experiences of exclusion related to their own specific multiple marginalized social identities. Our intercategorical analysis demonstrated how these experiences of differential treatment could be mechanistically mediated by aversive and systemic discrimination. Participants reported the impact these experiences had on their learning in various ways, including feeling “very strange” (P5) or an “outsider” (P1) at being “left out” (P3); feeling “frustrated”(P6) and “self conscious” (P11) that they have to “know more or come in more prepared” than their peers (P4); “doubted” (P12) and “questioned” around their expertise; “bothered,” (P12) “embarrassed” (P6) and “humiliated” P6) at being mistaken for their peers or other providers; and “uncomfortable”, “very worried” (P7) or “disappointed” (P9), “isolated” (P11), “very different”, or “annoyed” (P10) by the ways the default procedures result in often unintentional exclusion.

All participants experienced differential treatment compared to their peers as downstream effects of systemic and aversive discrimination in intercategorically intersectional ways. For example, participant 13 experienced differential treatment due to microaggressions where, as a racialized woman, her expertise was questioned in ways that her non-racialized male peers were not, which could mechanistically be explained by aversive discrimination;

“*I’ve noticed that the nurses will treat me [as a woman] differently compared to my male co-residents. If I ask for something, they oftentimes will question everything that I’m doing. Even if my co-resident is the same [junior] level of training, but is male, they will take them seriously – they don’t ask questions” –* Participant 13, related to being a racialized woman

And from the misalignment in the performativity of stated program EDI priorities and her own learning experiences.

*“I think the people that are in charge do care about EDI and are trying to make a difference…[but] the people that are not on board are not going to say anything… they will not help with [EDI] initiatives”* – Participant 13, related to being a racialized, low SES woman

Systemic discrimination contributed to her experiences of social exclusion and a silencing of her experiential expertise related to her low SES background due to how a supposedly “neutral” medical culture reflects high SES norms as normalized conversation about vacations.

*“In medicine, for example, everyone talks about, oh, they love to travel, and they talk about all of these places that they’ve travelled. And it’s hard because you realize that you don’t have any common ground to talk about. Because I haven’t travelled to all these different countries because we were just struggling to put food on the table*” – Participant 13, based on low SES background

*““Is this a reportable case to CAS?” One of my classmates, who was very well off… it was very black and white for him: “Yeah, this person is an awful parent because you can’t leave a six- year-old alone. This is so neglectful.” And I didn’t say anything at the time, but…my parents left me alone when I was four until nine, because they couldn’t afford a babysitter or daycare. I realised, oh, I shouldn’t talk.. about my background.” –* Participant 13, related to low SES background

Participant 13’s experience of subordination based on being a racialized, low SES woman contributed to her challenge with being perceived as a legitimate expert or peer by coresidents, supervisors, and patients alike. Participant 13’s struggle is a representative example of how other participants experienced the impact of systemic and aversive discrimination in intercategorically intersectional ways.

### Compensatory Strategies for Struggling with Discrimination

Multiple participants who identified as racialized woman reported several compensatory strategies for struggling with discrimination. They described performing to the expectations of the dominant culture to have their expertise and legitimacy as physicians recognized. These strategies included citing literature for their management suggestions, dressing in more formal clothing, introducing themselves to patients and care team members by their role, speaking more assertively, or intentionally attempting to speak English with a North American accent to appear closer to being white, Canadian, and male.

*“I try to have really more powerful looking work dresses or work outfits and stuff like that because I felt like it gave me a little bit more authority or people would take me a little bit more seriously” –* Participant 1, based on being a racialized woman

*“I found that when the male student brought up a change or add on to their management plan, it would sort of just be his word of mouth. And I had to actually bring in facts and papers.”* – Participant 7, based on being a racialized woman

“*Because I was raised by parents who spoke with an accent, I felt I was taken more seriously if I spoke without an accent, maybe more similarly to people around me, that I would be taken more seriously. I don’t like that about myself…But I don’t know if my white counterparts, for example, had to work harder to have their speech really articulate*.” – Participant 6, based on being a racialized woman

Despite these strategies, participants reported varying success in the learning environment. Other participants reported that the marginalizing effect attributed to their non-dominant identities could not be overcome by leveraging their own dominant identities, such as having extensive past training or physician family members.

*“I had family here who were doctors, so I had some guidance…[But we] don’t feel like you’re good enough…We have the knowledge, we’ve done the training, we’ve done much more in terms of medical school in terms of medical expert content…so the foundation of knowledge is there, but the culture of us being soft spoken and then [brown, Muslim] people of colour, it’s the same through all of that.” –* Participant 11, based on being a racialized internationally trained man

## Discussion

Our study highlights multiple empirical real-world examples of unequal social relations in the learning environment between learners with intersectionally marginalized backgrounds – regardless of their specific marginalized intersectional identities – and those with more dominant backgrounds. By applying an intercategorical intersectional approach, we demonstrate how the simultaneous processes of being racialized, gendered, or otherwise othered based on each participants’ unique social identities were the mechanisms that led to the reproduction of unequal social relations, and could be mechanistically accounted for by systemic and aversive discrimination. Moreover, participants reported various compensatory strategies to overcome this experience of differential treatment by attempting to perform to the dominant culture, with variable success. These findings resonate with the intersectional studies to date, which have demonstrated how the training experiences of students with intersectionally marginalized identities are different from that of their peers. Our study adds to this literature by using an intercategorical intersectional approach to identify the mechanisms for why students continue to experience discrimination despite institutional efforts. These findings can be used by residency training programs to detect, track, and intervene on more subtle forms of discrimination in the learning environment and meaningfully address inequities to ensure fairness in learning experiences (Table 2).

**Table 2:** Experiences Perpetuating Unequal Social Relations, Their Mechanism, and Potential Solutions.


PARTICIPANT EXPERIENCE OF FEELING LESSER THAN THEIR PEERS	MECHANISM	POTENTIAL SOLUTIONS

Having their professional and personal identities miscategorized in relation to their marginalized identities	Aversive Discrimination – Microaggressions as a result of implicit biases	Using real examples of micro-aggressions to develop and offer case-based education to both faculty and learners to bring attention to the aversive racist practices operating in their program. These educational interventions could encourage critical reflection practices to reduce bias and prejudice in daily interactions. This example is grounded in empirical studies that demonstrate when aversive racists are made aware of their bias, they reduce their biased behaviours [71]. This intervention is effective because the same studies show that it does not bias the behaviours of those who are already not aversively racist [71].

Participant experiences of microaggressions and other discrimination is mismatched from stated programmatic commitments to EDI	Aversive Discrimination – Supervisors and peers seemed unaware of acting on their implicit bias, in group favouritism	Using a hidden curricular framework to identify misalignments between the formal, informal, and hidden curricula and designing educational interventions to address identified policies and practices that generate misalignments [61]. The interventions above are one such example. Other potential solutions include: ensuring that pathways for reporting learner mistreatment are clear, and program responses are meaningful and accountable [69]; ensuring fairness in selections, promotions, and awards processes [62,64,65,66]; addressing inequities in programmatic assessment that may result from aversive discrimination [63,66,68]; the ongoing programmatic tracking of experiences of inclusion and exclusion in all the above categories to generate the empirical evidence for ongoing educational reform [75].

Exclusion from dominant social groups	Aversive Discrimination – Social dominance, in-group favouritism	The residency program invites regular anonymous feedback on resident experiences of inclusion, including examples of what practices and in what contexts these practices generate experiences of belonging or exclusion. Using a standing agenda item educators review this feedback and make adjustments to programming and community building activities that explicitly frame the residency body as an in-group and promote interaction between all residents in the program [72].

Experiencing exclusion because of social events that centre alcohol consumption and holiday scheduling that makes it much more challenging for participants to celebrate cultural holidays or observe religious practices important to them	Systemic Discrimination – the program centers dominant group cultural practices and norms	The residency program encourages different social activities. While the consumption of alcohol is not eliminated, care is taken so that it is not offered in all social activities [77]. The program pro-actively identifies cultural and religious holidays that are important to trainees and shares programmatic mechanisms for requesting that time off [77].

Experiencing exclusion related to perceived censorship related to taboo topics and the neutrality of medical culture	Systemic Discrimination – the professional culture of medicine	Similar to above, case-based educational interventions with real-life examples are used to foster critical consciousness in faculty and learners around their own positionality and how that impacts how they relate to each other and their patients in the learning and work environments. Within the context of this study, exemplar case scenarios could include examples of classism impacting learners or patients or Islamophobia. Examples should be specific to experiences of discrimination and censorship that are relevant and specific to each learning environment.


Importantly, although study participants did not share examples of overt or intentional discrimination, this does not mean that learning environments are free from such forms of exclusion. In fact, ongoing overt discrimination experienced by residents is well documented [5,55,56]. However, the lack of overt discrimination could reflect the incremental success of EDI institutional efforts and shifting societal values, which have made outwardly discriminatory views less socially unacceptable both within and outside of the medical profession. This possibility resonates with the broader sociological literature on how modern racism has become more implicit and structural [57,58,59,60]. Given the evolution of how discrimination shows up in the learning environment, our study findings highlight important mechanisms for how modern discrimination impacts learner training experiences in ways that are less obvious to the often unintentional perpetrators, but nevertheless contribute to experiences of differential treatment for those from marginalized backgrounds.

Moreover, the mismatch noted by participants between a program’s stated EDI values and their experiences in the learning environment can also help to explain why learning environment inequities have persisted despite invested institutional efforts to advance EDI in the learning environment. This mismatch is best described as a hidden curriculum effect [61]. The formal curriculum states that equity, diversity, and inclusion are important and valued, but these stated values are misaligned with the ways that teaching and learning occur in the learning environment, and were experienced as performative by participants. This finding highlights an important opportunity for addressing a manifestation of aversive discrimination through continuous quality improvement, faculty development efforts, and other culture change initiatives. These efforts should ensure that symbolic gestures and messaging, such as emails celebrating diversity, are consistent with other educational practices in admissions, promotions, and assessment processes in the learning environment [62,63,64,65,66,67,68,69].

While the role that systemic discrimination plays in perpetuating inequities has been acknowledged and explored in medical education, aversive racism in medical education has been less explored. Evidence-based interventions on aversive discrimination involve intervening on the underlying, otherwise normal, socio-cognitive processes. Research is mixed on effectiveness of directly confronting implicit biases, with concerns for paradoxical rebound effects [70,71]. Structural interventions to reduce biases or to resist biasing influences, harness an aversive racist’s egalitarian motives, or reframe what counts as an ingroup to address in group/out group dynamics have been shown to be effective [70,71,72,73,74]. Additionally, making aversive racists aware of their implicit prejudice prior to an activity has been shown to effectively reduce their bias; this could be implemented for residency selection and evaluation processes [71]. For example, describing all residency applicants as future colleagues during ranking discussions could reframe an otherwise heterogenous group as part of a medical profession in-group; this could interrupt aversive discrimination in selections processes, while still leaving space for a race-conscious approach to admissions (Table 2). Interventions intending to address exclusion and discrimination in the learning environment require ongoing study given the evolving and dynamic nature of systemic and aversive discrimination [75].

This study had several limitations. We were only able to theorize the mechanisms contributing to unequal social relations based on the marginalized social identities that were most represented in our study population at a single institution: mostly female, racialized as brown or black, internationally trained, and/or Muslim. Other social identities missing from our study population may contribute to learner experiences in different ways and require further study. While some participants occupied dominant social identities (i.e. white passing, health professional), when comparing their experiences to others, they were more likely to see themselves as different from what they perceived as the dominant social identities in their context. The sensitivity of the topic also limited our ability to recruit more actively. Despite these limitations, we were able to achieve adequate information power. Though we did not methodologically set out to find a representative sample or produce findings that are generalizable, the empirical evidence linking exclusion experiences to aversive and systemic discrimination as mechanisms for reproducing systemic inequities resonates with local institutional data [76], medical education literature [6,77], and beyond [6,57,58,59], suggesting that these phenomena are relevant and transferable beyond our institution to other Canadian and American residency training programs.

Generating empirical evidence for how aversive and systemic discrimination contribute to resident exclusion experiences is important because it highlights mechanisms that have received some scholarly attention but have not yet been the major focus of EDI interventions, especially in the current global climate where EDI programs are being re-evaluated. The explanatory power of covert mechanisms that perpetuate intersectional inequities, such as those described in our paper, are helpful for understanding why residents continue to report discrimination experiences despite programmatic or institutional efforts and are important for generating interventions to reform the learning environment. If we are to ensure that all learners are treated fairly in learning environments, we must identify and address systemic and aversive discrimination to disrupt the reproduction of unequal social relations.

## Previous presentations

The abstract of this article was presented at the Association of Medical Education in Europe Conference, the International Congress on Academic Medicine, and the International Conference on Residency Education.
